# Proteomic profiling of whole-saliva reveals correlation between Burning Mouth Syndrome and the neurotrophin signaling pathway

**DOI:** 10.1038/s41598-019-41297-9

**Published:** 2019-03-18

**Authors:** Guy Krief, Yaron Haviv, Omer Deutsch, Naama Keshet, Galit Almoznino, Batia Zacks, Aaron Palmon, Doron J. Aframian

**Affiliations:** 1 Institute of Dental Sciences, The Hebrew University, Jerusalem, Israel; 2 Sjogren’s syndrome Center, Department of Oral Medicine, Sedation and Maxillofacial Imaging, Faculty of Dental Medicine, The Hebrew University, Hadassah Medical Center, Jerusalem, Israel; 3 Salignostics Ltd., Jerusalem, Israel

## Abstract

Burning mouth syndrome (BMS) is characterized by a spontaneous and chronic sensation of burning in the oral mucosa, with no apparent signs. The underlying pathophysiological and neuropathic mechanisms remain unclear. Here, we attempt to elucidate some of these mechanisms using proteomic profiling and bioinformatic analyses of whole-saliva (WS) from BMS patients compared to WS from healthy individuals. Qualitative and quantitative proteomic profiling was performed using two dimensional gel electrophoresis (2-DE) and quantitative mass spectrometry (q-MS). In order to improve protein visibility, 21 high abundance proteins were depleted before proteomic profiling. Quantitative proteomic analysis revealed 100 BMS specific proteins and an additional 158 proteins up-regulated by more than threefold in those with BMS. Bioinformatic analyses of the altered protein expression profile of BMS group indicated high correlations to three cellular mechanisms including the neurotrophin signaling pathway. Based on this finding, we suggest that neurotrophin signaling pathway is involved in the pathophysiology of BMS by amplifying P75NTR activity, which in turn increases neural apoptosis thereby reducing sub-papillary nerve fiber density in the oral mucosa.

## Introduction

The diagnosis of burning mouth syndrome (BMS) is based on an intraoral burning or dysaesthetic sensation, recurring for more than two hours per day for at least 3 months, without clinically visible causative lesions.^1,2^. BMS predominantly affects postmenopausal women, with an overall prevalence ranging from 0.7% to 4.9% in the general population^3^. Patients may also exhibit xerostomia, dysesthesia and altered taste. Previous studies suggest a multifactorial etiology with oral, systemic and psychological factors^4–6^. BMS was classified in the International classification of Headache Disorders^7^ in the “Painful cranial neuropathies and other facial pains” section. Studies also suggest an association between BMS and neuropathological disorders, such as neural fiber loss in oral tissues^8^^,^^9^ salivary and somatosensory abnormalities^10^, reduced blink reflexes similar to Parkinson disease^11,12^, and peripheral nerve degeneration^13–15^.

The diagnostic potential of whole-saliva (WS) to reflect, detect and monitor physiological and pathophysiological conditions has been discussed in the literature^16^. WS comes from a variety of sources: salivary gland secretions, serum and serum derivatives, microbiota, oral mucosal cell debris, and nasal and gastrointestinal fluids^17^ which mix in the oral cavity. Proteomic profiling of WS is reliable and well established for the detection of up- or down-regulated proteins in particular diseases^18–21^. Based on proteomic profiling, and further bioinformatic analyses our understanding of the pathophysiology of diseases including BMS may improve^22,23^. Recently, 3,000 proteins have been identified in WS^24^ and proteomic profiling demonstrated altered protein expression in oral diseases such as oral squamous cell carcinoma^25^, Sjögren’s syndrome^18^, periodontitis^26^ as well as in neurological conditions such as Alzheimer disease^27^ and other central nervous system (CNS) diseases^28^. We decided to examine the proteomic profile of WS from BMS patients to determine whether changes exist and to see if these changes enhance our understanding of the pathophysiology of BMS. High abundance proteins (HAP), hamper proteomic analysis of WS, as previously described^29–31^. These highly expressed proteins include salivary alpha amylase isoforms (sAA), accounting for ~60% of the total protein^32,33^, and albumin isoforms forming ~20% of the total protein^30^. In other words, approximately 80% of the WS proteins come from a few HAP^31^ which obscure visualization of other proteins. Several HAP depletion methods have been developed^29–31,33^ and tested. In a previous study, we demonstrated that combining three HAP depletion protocols produces optimal depletion and maximizes protein visualization (and named the method enriched multiple depletion; “EMD”^30^). EMD integrates an enzyme substrate column and two immune-depletion kits and enables the depletion of 21 HAP including sAA and albumin isoforms. In this study EMD was used on WS from BMS patients and healthy controls, 2-DE and qMS analyses were then performed and the results were further analyzed with bioinformatic tools. Unique clusters of protein complexes were detected, correlating with the involvement of neuropathic mechanisms in BMS.

## Materials and Methods

Whole saliva (WS) was collected from 20 BMS patients, and 20 healthy volunteers for both pooled and individual analysis, as detailed in the Supplementary Table S3. Exclusion criteria were smoking, pregnancy, lactation, low levels of folic-acid and/or vitamin B-12, presence of oral lesions, local infections and diabetes.

### WS sample collection and pretreatment

The complete unstimulated whole saliva (WS) accumulation protocol was approved by the Ethical Committee of Hadassah Medical Center, Jerusalem, Israel. Research was performed in accordance with relevant guidelines/regulations; informed consent was obtained from all participants.

Samples were collected in pre-calibrated tubes using the previously described spitting method^34^. Samples were immediately placed on ice and then centrifuged at 14,000* g* for 20 min at 4 °C to remove insoluble materials, cell debris and food remnants^29^.

### High abundance protein (HAP) depletion

21 HAP were depleted using the enriched multiple depletion EMD method which combines sAA depletion^30^, Alb and IgG immune-depletion (ProteoPrep® Immuno-affinity Depletion Kit, Sigma-Aldrich, St Louis, MO) and the ProteoPrep® 20 plasma immune-depletion kit, Sigma-Aldrich, St Louis, MO). For further details regarding EMD see Krief *et al*.^30^. See Table S4 for the complete list of depleted HAP.

### Determination of Protein Concentration

Protein concentration was determined using the Bio-Rad Bradford protein assay (Bio-Rad, Hercules, CA) as previously described^35^.

### SDS-PAGE and densitometry

**Figure 1:**
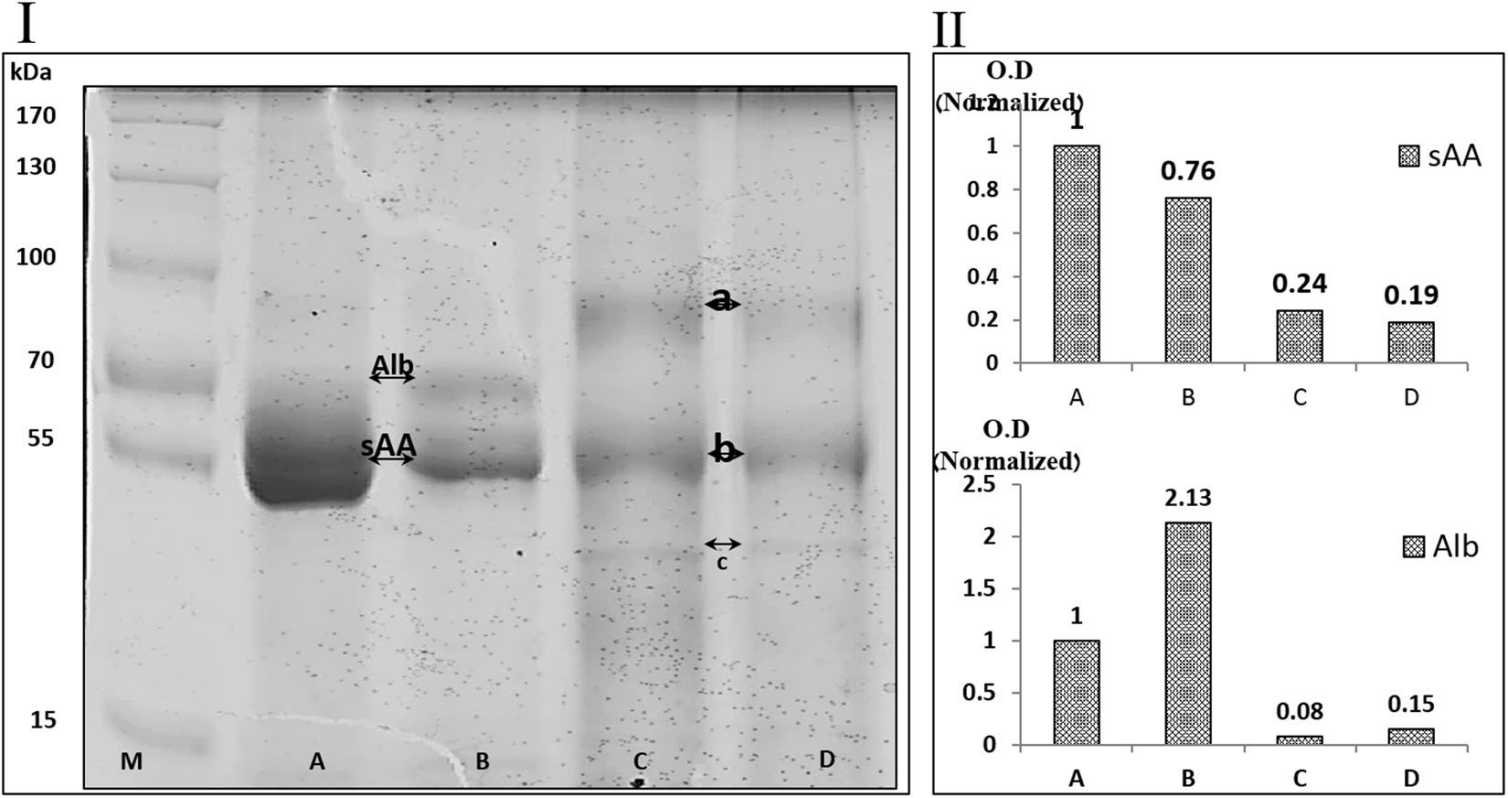
SDS-PAGE and densitometry of WS from healthy controls and BMS groups before and after HAP depletion.

10 µg protein samples pooled from the WS of BMS and healthy individuals, before and after HAP depletion, were separated on an 8% acrylamide mini gel and stained using Coomassie Brilliant Blue, as described^33^. Following band normalization, band patterns were measured and compared using ImageJ software (Fig. 1).

### Two-dimensional gel electrophoresis (2-DE)

For analytical gels, following HAP depletion, 100 µg protein samples pooled from healthy and BMS groups, were rehydrated and then underwent iso-electrical focusing on 18 cm, pH 3–8, non-linear (NL) iso-electrical focusing strips. The second dimension was separated as previously described^18^. Gels were silver stained using the SilverQuest kit (Invitrogen, Carlsbad, CA, USA), as previously described^36^.

### Imaging and statistical analysis

Gels were scanned using a computer GS-800 calibrated densitometer (Bio-Rad) and spots were detected and measured using PDQuest software V 6.2.0 (Bio-Rad). In order to minimize the limitations of 2D gel analysis resulting from gel to gel variation, and lack of uniformity in staining, the samples were exposed to the same conditions simultaneously for the first and second dimensions. Normalization using PDQuest was performed using the total density in image method to semi-quantify spot intensities and to minimize staining variation between gels.

### Quantitative MS (q-MS)

HAP depleted pooled protein samples (10 µg) from the study groups were analyzed by q-MS. 8 M Urea, 100 mM ammonium bicarbonate and reduction with 2.8 mM DTT (60 °C for 30 min) were used for proteolysis. Proteins were then modified with 8.8 mM iodoacetamide in 100 mM ammonium bicarbonate (in the dark, at room temperature for 30 min) and digested in 2 M Urea, 25 mM ammonium bicarbonate with modified trypsin (Promega) at a 1:50 enzyme-to-substrate ratio, overnight at 37 °C. A second digestion was performed by repeating the procedure for 4 hours. The tryptic peptides were then desalted using C18 tips, dried and re-suspended in 0.1% Formic acid. The peptides were resolved by reverse-phase chromatography on 0.075 × 200-mm fused silica capillaries (J&W) packed with Reprosil reversed phase material (Dr. Maisch GmbH, Germany). The peptides were then eluted with linear 214 minutes gradients of 7 to 40% and 8 minutes at 95% acetonitrile with 0.1% formic acid in water at flow rates of 0.25 μl/min. Mass spectrometry was performed by an ion-trap mass spectrometer (Orbitrap, Thermo) in a positive mode using a repetitively full MS scan followed by collision induced dissociation (CID) of the 7 most dominant ions selected from the first MS scan. Wash runs and a blank injection were performed between samples to prevent cross contamination. The mass spectrometry data was analyzed using MaxQuant 1.2.2.5 software (Mathias Mann’s group), by searching against the human section of the Uniprot database, and quantified by label free analysis using the same software. Statistical analysis was performed using Perseus software (Mathias Mann’s group).

Proteins were sorted into 7 groups for bioinformatic analyses, based on BMS to healthy expression ratio.

### Bioinformatics

Data from q-MS was analyzed using three online bioinformatics tools. First, we performed “functional annotation clustering” using DAVID bioinformatics resources 6.7 online version (http://david.abcc.ncifcrf.gov). Next we analyzed the association network of the proteins found only in BMS using “STRING” online database (http://string-db.org/), which maps and scores protein-protein interactions. The protein association map was then intersected against the “KEGG PATHWAY Database” (http://www.genome.jp/kegg/pathway.html) to match protein complexes to relevant pathways. Finally, the participating proteins in the neurotrophin signaling pathway were matched to the BMS identified proteins using the “KEGG PATHWAY database” (http://www.genome.jp/kegg-bin/show_pathway?hsa04722)^37^.

## Results

### SDS-PAGE

Figure 1 shows the band pattern of WS proteins (SDS-PAGE) before and after HAP depletion in BMS and healthy controls. The bands representing sAA (55 kDa) and Alb (69 kDa) are labeled in lanes A and B based on previous protein identifications^29^. Densitometry analysis (part II) of sAA and Alb bands with normalized O.D. shows that the band pattern of lane B was similar to lane A, and sAA and Alb were the most dominant proteins. Densitometry analysis of lane B indicated a 24% decrease in the O.D. of the sAA band and a 113% increase in O.D. of the Alb band. The band pattern of lane C showed a reduction of 76% in the O.D of the sAA band and a 92% decrease in the O.D. of the Alb band. The band pattern of lane D was similar to lane C with O.D. reductions of 81% and 85% in sAA and Alb respectively. In addition to the decreased O.D. of sAA and Alb in the depleted lanes, new bands (marked a, b and c) became visible.

### 2DE

**Figure 2:**
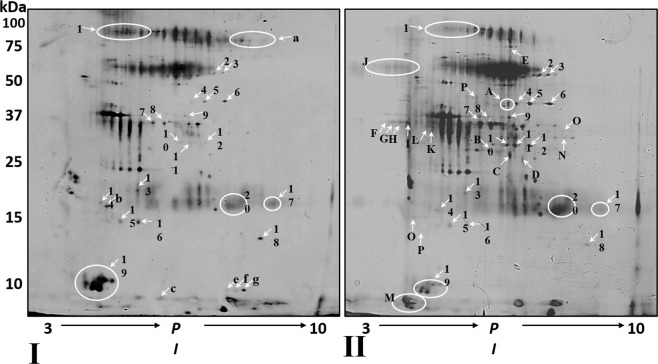
2-D gel electrophoresis of pooled, HAP depleted WS from healthy controls and BMS groups.

Figure 2 shows 2DE gels from pooled healthy (I) and BMS (II) samples after HAP depletion. Qualitative proteomic comparison revealed that 237 spots were detected in gel I and 313 spots were detected in gel II. Spot matching analysis revealed that 6 spots were only found in gel I whereas 82 spots were only detected in gel II. 231 matched spots were observed, 12 had a more than fivefold increase in O.D. in gel II (spots 2–12, 16), and 8 had a decrease of more than fivefold in O.D. (spots 1, 13–15, 17–20) in gel II compared to gel I. Comparison between the spot patterns of the gels highlights that main differences between the healthy and the BMS patients come from a large number of spots only seen in gel II.

### Quantitative MS

**Figure 3:**
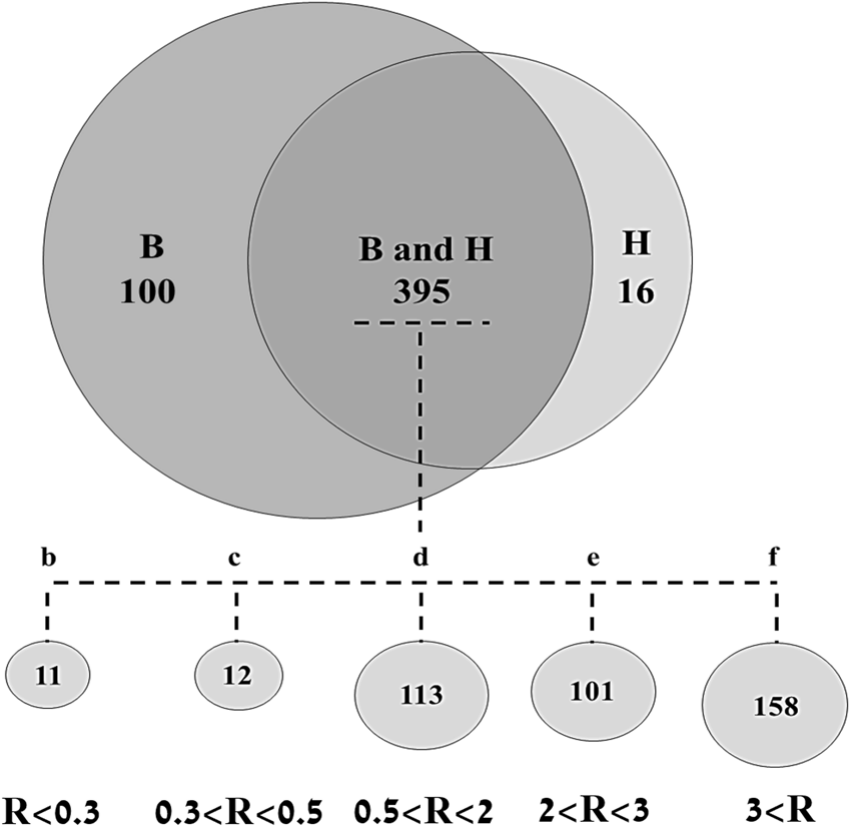
Venn diagram summaries of q-MS identified proteins in healthy controls and BMS.

Quantitative proteomic comparison of pooled WS from healthy controls and BMS patients after HAP depletion was performed using q-MS (Tables 1a to 1g in supplementary). 511 proteins were identified and quantified in healthy controls and BMS samples. For analytical purposes, proteins were grouped by their BMS to healthy control expression ratio (Fig. 3). Group a included 16 proteins only identified in healthy controls (Table 1a). Group b included11 proteins with expression levels at least 3 times higher in the healthy controls than in BMS (Table 1b). Group c contained 12 proteins with a 2–3 fold increase in expression in healthy controls compared to BMS (Table 1c). Group d included 113 proteins with similar expression in both groups (Table 1d). Group e contained 101 proteins with 2–3 fold increase in expression in BMS compared to controls (Table 1e). Group f contained 158 proteins with more than a threefold increase in expression in BMS compared to controls (Table 1f). Group g contained 100 BMS specific proteins (Table 1g).

### Functional annotation clustering

**Figure 4:**
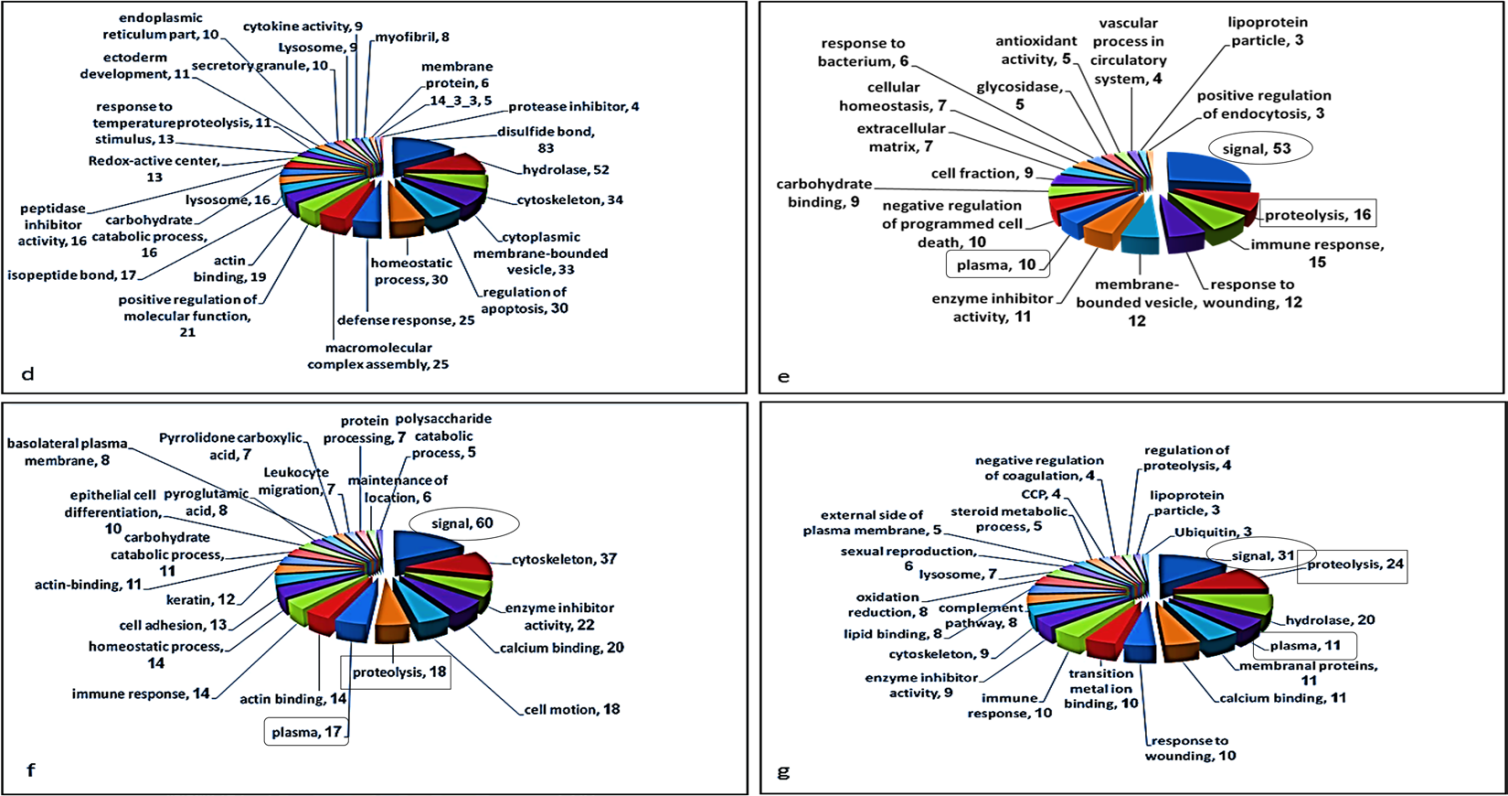
Functional annotation clustering. Ratio Groups (**A**–**D**) were analyzed using functional annotation clustering (DAVID Bioinformatics Resources 6.7). The functional clustering pattern was compared between the ratio groups. Clusters found in more than one ratio group (cut off 10 proteins) are indicated.

Functional annotation clustering was performed using DAVID bioinformatics database (http://david.abcc.ncifcrf.gov). Analysis was performed on the proteins from group d - g (Table S2 (supplementary) and Fig. 4). Groups a, b and c were excluded due to the low number of proteins in each group. Twenty-five functional clusters were observed in group d, 11 in common with other groups and 14 unique clusters. Seventeen functional clusters were observed in group e, 8 shared with other groups and 11 unique clusters. Group f had 10 shared and 10 unique clusters. Twenty-three functional clusters were observed in group g, 13 shared and 10 unique clusters. Comparison between the functional clusters of the groups shows that the clusters “signal”, “plasma” and “immune-response” were all observed in groups e, f and g (up-regulated in BMS) with more than 10 attributed proteins in each cluster. However, none of these functional clusters were observed in group d.

### Protein association network

**Figure 5:**
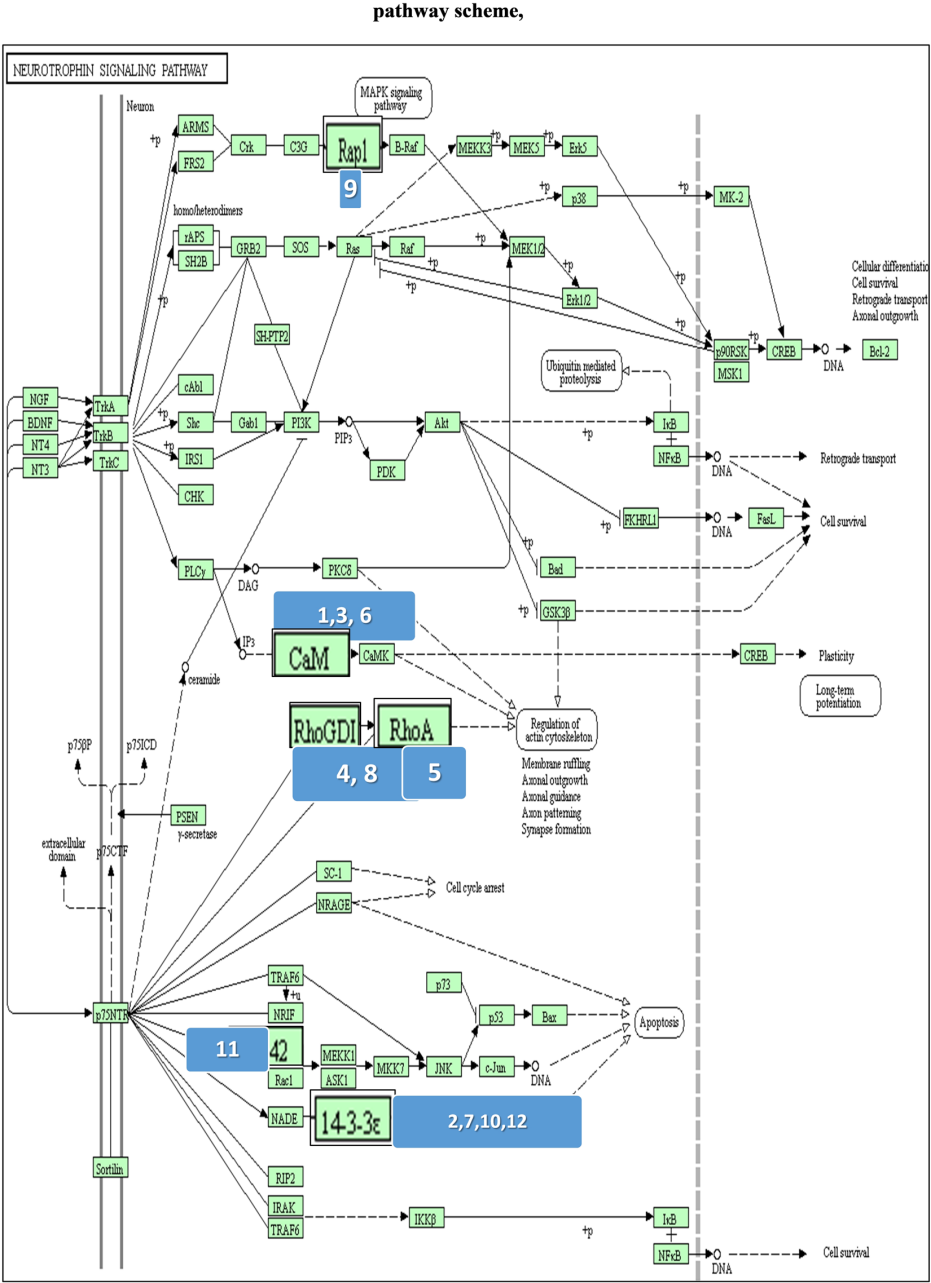
Pathway scheme with marked involved proteins^37^. BMS identified proteins and proteins involved in the neurotrophin signaling pathway were matched by DAVID Bioinformatics Resources. Up and down regulated proteins in BMS are enlarged.

The protein association network maps protein complexes and provides a system-level understanding of biological mechanisms. Therefore, we analyzed the functional association network of the proteins in group g (proteins with the most significant connection to BMS). Figure 5s (supplementary) shows high-confidence protein-protein interaction network of between the proteins g, generated by STRING 9.1 database (http://string-db.org). In order to match the proteins complexes to biological functionalities, we intersected the results with the GenomeNet database (http://www.genome.jp). This indicated three protein complexes with physiological mechanisms (complexes were based on 2 proteins or more). Cluster 1 included 8 associated proteins, participating in complement and coagulation cascades, cluster 2 included 7 associated proteins, participating in the proteasome complex, cluster 3 included two associated proteins, involved in the neurotrophin signaling pathway (Fig. 5s).

### BMS and neurotrophin signaling pathway proteins match

In order to establish a possible link between BMS and the neurotrophin signaling pathway, we matched the proteins from groups d-g with the proteins known to participate in this pathway. Figure 5 is a schematic view of the neurotrophin signaling pathway with BMS matched proteins marked by ellipses. Table 1 lists the 12 BMS proteins, involved in the neurotrophin signaling pathway, with their BMS to healthy expression ratios. Examination of the BMS to healthy expression ratio of these 12 proteins, showed 2 proteins only identified in BMS, 9 up-regulated proteins in BMS (by more than 1.82 fold), and one down-regulated protein (BMS to healthy expression ratio of 0.62). When examining the location of these proteins in the neurotrophin signaling cascade, 8 were down-stream to the p75 receptor (numbered as 2, 4, 5, 7, 8, 10, 11 and 12), and 4 were down-stream to Trk receptor proteins (numbered as 1, 3, 6 and 9).

**Table 1:** list of involved proteins in neurtrophin signaling pathway.

No	Protein ID	Accession	BMS/Healthy
1	Calmodulin	P62158	Only in BMS
2	14-3-3 protein eta	Q04917	Only in BMS
3	Calmodulin-like protein 3	P27482	6.46
4	Rho DDP-dissociation inhibitor 1	P52565	5.7
5	Transforming protein RhoA	P61586	4
6	Calmodulin-like protein 5	Q9NZT1	2.86
7	14-3-3 protein epsilon	P62258	2.65
8	Rho GDP-dissociation inhibitor 2	P52566	2.16
9	Ras-related protein Rap-1b	P61224	1.96
10	14-3-3 protein	P31946	1.96
11	Cell division control protein 42 homolog	P60953	1.82
12	14-3-3 protein theta	P27348	0.62

A detailed list of involved proteins in neurtrophin signaling pathway, together with their accession numbers and expression ratio.

## Discussion

The pathophysiological mechanisms of BMS are poorly understood. We employed qualitative and quantitative proteomic approaches, coupled with bioinformatic analyses of WS from BMS patients to increase our understanding of this condition. In order to obtain high proteomic resolution for sensitive detection of low abundance proteins, 21 HAP were depleted from the samples including the most abundant WS proteins sAA and Alb. The depletion methodology employed has been successfully used previously and improves visualization of WS proteins^30^. Figure 1 shows the protein pattern before and after HAP depletion, following SDS-PAGE and densitometry analysis and clearly demonstrates that HAP depletion is essential if new proteins are to be detected, such as bands a, b and c in lanes C and D. The importance of HAP depletion to improve visualization of lower abundance proteins has been noted for WS and other body fluids^30,38^. The differences between the protein profiles of healthy controls and BMS were observed by qualitative analysis of 2-DE and similar to those observed by q-MS analysis, with 100 proteins unique to BMS and 259 with expression levels increased by more than twofold in BMS compared to healthy controls. Several bioinformatic tools were used to elucidate the pathophysiological significance of our findings. The identified proteins were divided into 7 groups based on their BMS to healthy expression ratios (Fig. 3 and Tables 1a–g). First, we mapped functional clusters of groups d, e, f and g using functional annotation clustering analysis, as shown in Table S2 and Fig. 4. Functional cluster comparison revealed several shared clusters in groups e, f and g, which were dissimilar to the cluster pattern of group d. The clusters “signal”, “proteolysis” and “plasma”, included ten or more proteins per cluster in groups e, f and g. However, these cluster were not observed (“signal” and “plasma”) or poorly observed (“proteolysis”) in-group d. These findings suggest that WS from BMS contains up-regulated proteins involved in signal transduction, proteolytic activity and inflammation. Considering that group g included proteins with the greatest overexpression in BMS, a system-level analysis by functional association network to discover protein complexes, was performed. We further intersected the association map against the KEGG pathway database to elucidate protein complexes involved in biological pathways, as shown in Fig. 5s. Three significant pathways (p-value < 0.005), matching the proteomic complexes were found: a complex of 8 proteins involved in “complement and coagulation” mechanisms, a complex of 7 “proteasome” subunits and 2 proteins involved in “neurotrophin signaling pathway”. These findings correlate to the functional annotation analysis (Fig. 4). In contrast to the first two mechanisms, the neurotrophin signaling pathway is specific to pain sensation^39,40^, and therefore may have a major role in BMS.

The neurotrophin protein family controls many aspects of neuronal survival, development and function in both the peripheral and the central nervous systems^41^. Furthermore, based on clinical trials on neuropathy and neurodegeneration, acute pain is a known side effect of neurotrophins^42^. In order to determine whether the neutrophin signaling pathway is involved in the pathophysiology of BMS, we searched for an overlap between the proteins identified by q-MS proteins in this pathway. Twelve matched proteins were detected (shown in Fig. 5), all were overexpressed in BMS except one (14-3-3 theta). Neurotrophins act via two neuronal membrane receptors: Trk tyrosine kinase receptor and p75 neurotrophin receptor (p75NTR)^43^. Trk signaling is regulated by a variety of intracellular signaling cascades, which eventually transmit signals with positive effects enhancing nerve survival and growth. p75NTR transmits signals with positive and negative effects, and the ratio of activity Trk/p75NTR is important in neural development, survival and apoptosis^44,45^.

From the 12 BMS matched proteins, 8 were down-stream to p75NTR and four were down-stream to Trk (detailed in Table 1). These differently expressed proteins in BMS tip the balance of the Trk/p75NTR cascades, causing a negative impact on neural development. It is possible that the characteristic pain sensation of BMS is related to amplification of p75NTR cascades. for instance, increased levels of calmodulin, 14-3-3 protein eta and Rho GDP-dissociation inhibitor 1 in BMS located downstream to p75TR, elevates P75NTR expression causing death of engrailed-deficient midbrain dopaminergic neurons by Erk1/2 suppression in mice. Moreover, an association between BMS and reduced endogenous levels in the putamen resulting in changed central nociceptive signal processing has been found^13^.

Taken together, bioinformatic analysis which compared q-MS profiling to the neurotropin signaling pathway supports the notion that increased neural apoptosis via amplified p75NTR activity in the neurotrophin signaling receptor, is involved in BMS pathophysiology and symptoms. Since no membrane proteins are secreted in saliva we assume that these proteins enter WS when mucosal cells lining the oral cavity are exfoliated, mainly from the tongue. This assumption is based on the clinical observation that the tip of the tongue is the area primarily affected in individuals with BMS, and this site is rich in filiform papillae. Braud *et al*.^46^, recently showed that electrogustometric thresholds of lingual fungiform and foliate gustatory papillae function increased significantly in BMS patients. Interestingly, lingual somatosensory axons that terminate in each filiform papilla express the p75 neurotrophin receptor (p75NTR)^47^. Future studies should use pure saliva, collected from individual glands e.g. by using a Carlson-Crittenden cup for parotid saliva collection in order to reduce contamination^48^.

A recent proteomic analysis of saliva from BMS patients found 3 biomarkers: alpha-enolase, IL-18, and KLK13 potentially associated with peripheral nerve damage and increased inflammation^49^. In our study, KLK18 protein levels were 3-fold higher in BMS patients (Table 1e), but IL-18 nor alpha-enolase were not detected. A possible cause for the discrepancy is different inclusion criteria e.g. dry mouth was not a criteria in the current study. Moreover, the lack of uniform diagnostic criteria for BMS may also contribute to different findings^50^. Another study found that cystatin SN was over-expressed in unstimulated saliva of BMS patients, and suggested a connection between cystatin SN levels and ongoing inflammation^51^. Similarly, we noted a 2.35-fold increase in cystatin SN levels in BMS samples compared to controls. Moreover, 8 proteins over-expressed in BMS were clustered as associated with “complement” pathways relating to inflammation. However, this general mechanism is associated with many conditions e.g. gingivitis. In contrast, the neurotrophin signaling pathway is more specific and correlated to pain in general and to BMS specifically by alternation of pathway activity toward neural apoptosis. In conclusion, proteomic and bioinformatic approaches were used to examine WS from healthy individuals and those with BMS. The proteomic profile of WS from BMS patients has distinctive characteristics. Comparison to healthy controls revealed over 250 up-regulated and unique proteins in BMS. Examination of these proteins using several bioinformatic tools highlighted links to biological mechanisms including the neurotrophin signaling pathway. Preliminary validations showed the same pattern in individual samples, and reinforced the correlation between BMS and the neurotrophin signaling pathway. Based on our findings, we suggest than an increase in p75NTR receptor activity, which mediates neural apoptosis may be involved in BMS, and may be connected to alterations in the dopamine pathway. Reduced concentrations of epithelial and sub-papillary nerve fibers among BMS patients have been observed^9^. However, to the best of our knowledge, this is the first study with proteomic evidence of increased neural apoptosis in BMS patients via altered expression of proteins in the neurotrophin signaling pathway. Further research is necessary to fully understand the mechanisms, and to determine whether BMS is an idiopathic neuropathic disease associated with apoptosis.

## Supplementary information

Supplementary tables

Figure 5s. Protein association network
