# Supplementary material for: Proteomic profiling of whole-saliva reveals correlation between Burning Mouth Syndrome and the neurotrophin signaling pathway
Source: Sci Rep. 2019 Mar 18;9:4794. doi: 10.1038/s41598-019-41297-9 (PMC6423135; doi:10.1038/s41598-019-41297-9)
Supplement: Supplementary file 1 — Supplementary tables [file 41598_2019_41297_MOESM1_ESM.pdf]

Proteomic profiling of whole-saliva reveals correlation between Burning Mouth Syndrome and the neurotrophin signaling pathway

Guy Krief[1,3], Yaron Haviv[2*], Omer Deutsch[1,3], Naama Keshet[2], Galit Almoznino[2] Batia Zacks[2], Aaron Palmon[2], Doron J. Aframian[1]

Table 1a. Proteins identified only in healthy

no'	*Protein ID*	*Accession no'*	*Mol. weight [kDa]*	*Sequence coverage Healthy [%]*	*Sequence coverage BMS [%]*	*Healthy/BMS Intensity ratio*
1	Desmoplakin	P15924	331.77	0.7	0	**4.81093E-06**
2	Lymphocyte-specific protein 1	P33241	37.191	3.5	0	**6.18735E-06**
3	Enhancer of rudimentary	P84090	12.259	15.4	0	**6.45161E-06**
4	gamma-glutamyltransferase K	P22735	89.786	1.3	0	**8.03084E-06**
5	Dihydropteridine reductase	P09417	22.408	5.2	0	**9.45716E-06**
6	Hemoglobin subunit delta	P02042	16.055	23.1	32.7	**1.15606E-05**
7	Tubulin beta chain	P07437	47.766	4.2	4.5	**1.27243E-05**
8	Titin	Q8WZ42	3816	0.1	0	**1.4935E-05**
9	Pigment epithelium-derived	P36955	46.312	4.8	0	**1.60989E-05**
10	Tenascin-X;Putative	P22105	456.19	0.4	0	**2.27827E-05**
11	Alpha-2-antiplasmin	P08697	54.565	3.9	0	**2.48793E-05**
12	Phosphatidylinositol-glycan	P80108	92.335	2	0	**2.64019E-05**
13	N-acetylmuramoyl-L-alanine amidase	Q96PD5	62.216	2.4	0	**2.99616E-05**
14	Cytosol aminopeptidase	P28838	56.166	3.7	0	**4.25423E-05**
15	Keratinocyte proline-rich protein	Q5T749	64.135	2.1	0	**8.39137E-05**
16	CapZ-interacting protein	Q6JBY9	41.261	2.8	0	**8.53825E-05**

Table 1b . Proteins with more than 3 fold increased expression in the healthy sample relatively to BMS

no'	*Protein ID*	*Accession no'*	*Mol. weight [kDa]*	*Sequence coverage Healthy [%]*	*Sequence coverage BMS [%]*	*BMS /Healthy intensity ratio*
1	Keratin, type I cytoskeletal 13	P13646	49.588	40.4	38.2	**0.0537**
2	LIM domain-binding protein 3	O75112	77.134	5.8	2.1	**0.1257**
3	F-box only protein 50	Q6ZVX7	30.847	3.3	10.2	**0.1577**
4	Glutaredoxin-1	P35754	11.776	39.6	7.5	**0.2068**
5	Adenylate kinase 2, mitochondrial	P54819	25.63	14.2	7.3	**0.2235**
6	Mucin-7	Q8TAX7	39.158	10.3	8.8	**0.2549**
7	ADP-sugar pyrophosphatase	Q9UKK9	24.327	11.4	5	**0.2726**
8	Kallikrein-6	Q92876	26.855	8.2	8.2	**0.2874**
9	Ribosyldihydronicotinamide dehydrogenase [quinone]	P16083	25.918	31.2	30.3	**0.2888**
10	Histone H3.2	Q71DI3	15.388	37.5	48.5	**0.2914**
11	Stress-70 protein, mitochondrial	P38646	73.68	1.9	1.9	**0.3025**

Table 1c. Proteins with 2 to 3 fold increased expression in the healthy sample relatively to BMS

no'	*Protein ID*	*Accession no'*	*Mol. weig ht [kDa]*	*Sequence coverage Healthy [%]*	*Sequence coverage BMS [%]*	*Healthy/BMS intensity ratio*
1	Nuclear transport factor 2	P61970	14.478	27.6	16.5	**0.3379**
2	Pyridoxal kinase	O00764	35.102	18.9	15.7	**0.3431**
3	Thymidine phosphorylase	P19971	49.955	21.2	7.5	**0.3478**
4	UMP-CMP kinase	P30085	22.222	11.2	6.6	**0.3493**
5	Submaxillary gland androgen-regulated protein 3B	P02814	8.1875	65.8	65.8	**0.3645**
6	Transitional endoplasmic reticulum ATPase	P55072	89.321	5.5	4.2	**0.4100**
7	Aminopeptidase B	Q9H4A4	68.168	5.4	7.2	**0.4438**
8	Stathmin	P16949	17.302	11.4	6.7	**0.4562**
9	Carboxypeptidase E	P16870	53.15	10.5	7.6	**0.4687**
10	EF-hand domain-containing protein D2	Q96C19	26.697	4.6	4.6	**0.4863**
11	Serine protease 27	Q9BQR3	31.939	9	9	**0.4905**
12	Ubiquitin-conjugating enzyme E2 variant 1	Q13404	16.495	8.2	8.2	**0.4968**

Table 1d. Proteins with equal expression level between healthy and BMS samples

no'	*Protein ID*	*Accesion no'*	*Mol. weight [kDa]*	*Sequence coverage Healthy [%]*	*Sequence coverage BMS [%]*	*Healthy/BMS intensity ratio*
1	Secretoglobin family 1D member 2	O95969	9.925	27.8	10	**1.99**
2	Involucrin	P07476	51.367	2.3	6.8	**1.95**
3	Interleukin-18	Q14116	22.326	7.3	8.3	**1.94**
4	Thioredoxin-dependent peroxide reductase, mitochondrial	P30048	25.838	15.5	5.9	**1.89**
5	Protein FAM3D	Q96BQ1	24.963	33	30.4	**1.87**
6	Glyoxalase domain-containing protein 4	Q9HC38	34.793	31.6	35.8	**1.83**
7	SH3 domain-binding glutamic acid-rich	Q9H299	9.3804	59.1	43.2	**1.76**
8	Calpastatin	P20810	69.749	2.8	2.8	**1.70**
9	6-phosphofructokinase, liver type	P17858	85.018	1.2	1.2	**1.68**
10	Serum paraoxonase/arylesterase 1	P27169	39.731	5.4	2.5	**1.64**
11	14-3-3 protein theta	P27348	27.764	3.7	7.8	**1.61**
12	Proteasome subunit beta type-2	P49721	22.836	9.5	13.4	**1.57**
13	Tropomyosin alpha-4 chain	P67936	28.521	24.6	26.2	**1.56**
14	Histone H2B type 1-M	Q99879	13.989	50.8	50	**1.56**
15	Carbonic anhydrase 3	P07451	29.557	5	5	**1.52**
16	Tetranectin	P05452	17.794	13.1	13.1	**1.50**
17	Interleukin-36 gamma	Q9NZH8	18.721	29	21.9	**1.49**
18	Cystatin-SA	P09228	16.445	75.9	73	**1.42**
19	Basic salivary proline-rich protein 3	Q04118	30.98	6.8	6.8	**1.41**
20	Ubiquitin thioesterase OTUB1	Q96FW1	28.05	7.9	3.7	**1.40**
21	Glutathione reductase, mitochondrial	P00390	56.256	16.5	14	**1.39**
22	Superoxide dismutase [Cu-Zn]	P00441	15.936	52.6	76	**1.39**
23	Kunitz-type protease inhibitor 1	O43278	52.373	10.6	15.9	**1.38**
24	Tropomodulin-3	Q9NYL9	20.79	9.1	9.1	**1.37**
25	GDP-L-fucose synthase	Q13630	21.843	11.6	6.1	**1.36**
26	Thrombospondin-1	P07996	129.38	0.9	2	**1.35**
27	Prothymosin alpha;Thymosin alpha-1	P06454	11.758	15.9	13.1	**1.28**
28	CD59 glycoprotein	P13987	11.985	33.3	33.3	**1.27**
29	Histone H1.2	P16403	21.364	9.9	9.9	**1.27**
30	PITH domain-containing protein 1	Q9GZP4	24.178	4.3	4.3	**1.27**
31	Tropomyosin beta chain	P07951	32.85	33.1	26.8	**1.26**
32	Glutamine synthetase	P15104	42.064	7	10.2	**1.26**
33	Plastin-3	P13797	65.632	7.7	12.1	**1.25**
34	Fibromodulin	Q06828	43.178	5.3	5.3	**1.24**
35	Keratin, type II cytoskeletal 6A	P02538	60.044	43.8	41.5	**1.22**
36		Q5T0I0	28.953	40.8	65.8	**1.22**
37	Interleukin-1 receptor antagonist protein	P18510	20.055	47.5	48.6	**1.22**
38	Cathepsin L1;Cathepsin L1 heavy chain	P07711	37.564	8.7	4.2	**1.21**

39		P00761	24.409	25.1	31.6	**1.20**
40	Neutrophil elastase	P08246	28.518	18.4	8.2	**1.20**
41	Cathepsin Z	Q9UBR2	33.868	21.1	17.2	**1.19**
42	Cystatin-C	P01034	15.799	67.1	63.7	**1.18**
43	Folate receptor alpha	P15328	29.819	49.8	49.8	**1.17**
44	S-formylglutathione hydrolase	P10768	31.462	7.4	7.4	**1.17**
45	ATP synthase subunit beta, mitochondrial	P06576	56.559	12.5	10	**1.15**
46	Basic salivary proline-rich protein 4	P10163	18.011	12.4	12.4	**1.15**
47	Coronin-1A	P31146	51.026	20.2	26	**1.14**
48	Macrophage-capping protein	P40121	38.498	9.2	15.5	**1.13**
49	Thymosin beta-10	P63313	5.0256	36.4	36.4	**1.13**
50	Histone H2A type 1-J	Q99878	13.936	14.1	14.1	**1.12**
51	Desmoglein-3	P32926	107.53	20.4	19	**1.11**
52	Poly(U)-specific endoribonuclease	P21128	46.872	11.2	14.6	**1.11**
53	Cytidine deaminase	P32320	16.185	39	49.3	**1.11**
54	Plasma retinol-binding protein(1-182)	P02753	22.944	20.6	28.6	**1.11**
55	Tubulin-specific chaperone A	O75347	12.855	32.4	24.1	**1.11**
56	Lamin-B1	P20700	66.408	3.1	3.1	**1.10**
57	Calcium-activated chloride channel	Q14CN2	101.28	2.4	2.4	**1.08**
58	Kallikrein-11	Q9UBX7	31.059	36.5	26.6	**1.06**
59	Ras-related protein Ral-B	P11234	23.408	5.8	5.8	**1.06**
60	Omega-amidase NIT2	Q9NQR4	30.608	25.7	19.9	**1.06**
61	Histone H1.5	P16401	22.58	9.3	9.3	**1.05**
62	Thioredoxin domain-containing protein 5	Q8NBS9	40.369	8.3	8.3	**1.05**
63	Phosphoglycerate mutase 1	P18669	28.804	38.6	48.8	**1.05**
64	Coactosin-like protein	Q14019	15.945	40.1	59.2	**1.05**
65	Gamma-glutamylcyclotransferase	O75223	18.398	40.4	40.4	**1.04**
66	Proteasome subunit alpha type-1	P25786	29.555	17.5	19.4	**1.04**
67	Fatty acid-binding protein, epidermal	Q01469	15.164	94.8	94.8	**1.03**
68	Macrophage migration inhibitory factor	P14174	12.476	22.6	17.4	**1.02**
69	Leucine-rich alpha-2-glycoprotein	P02750	38.177	37.5	35.4	**1.01**
70	Ig kappa chain V-I region HK102	P01602	12.768	31.6	31.6	**1.00**
71	Thioredoxin reductase 1	Q16881	54.546	5	5	**1.00**
72	Zinc-alpha-2-glycoprotein	P25311	59.998	42.6	41.5	**0.97**
73	Heat shock protein beta-1	P04792	15.075	0	0	**0.96**
74	Eosinophil lysophospholipase	Q05315	13.92	41.3	40.5	**0.96**
75	Alpha-1B-glycoprotein	P04217	81.04	2	3.5	**0.88**
76	Lactotransferrin	P02788	192.75	3.2	6.9	**0.86**
77	BPI fold-containing family A member 2	Q96DR5	83.868	39.3	44.8	**0.59**
78	Cornulin	Q9UBG3	28.433	13.7	33.3	**0.58**
79	ERO1-like protein alpha	Q96HE7	21.731	46.9	55.6	**0.58**
80	hydrolase domain-containing protein	Q96IU4	34.258	60.4	61.4	**0.58**
81	UPF0556 protein C19orf10	Q969H8	22.782	50.2	38.5	**0.57**
82	Serpin B4	P48594	16.453	47.9	47.9	**0.57**
83	Gelsolin	P06396	9.9937	68.1	63.7	**0.57**
84	Protein FAM3B	P58499	26.021	9.2	16.2	**0.57**
85	14-3-3 protein sigma	P31947	20.96	12.8	16.8	**0.57**

86	Protein disulfide-isomerase	P07237	39.42	67.9	67.3	**0.56**
87	Cathepsin D	P07339	46.247	38	40	**0.56**
88	Cocaine esterase	O00748	120.71	21.8	26.8	**0.56**
89	Twinfilin-2	Q6IBS0	32.949	8.4	17.8	**0.56**
90	L-lactate dehydrogenase B chain	P07195	65.408	7.9	8.2	**0.56**
91	Resistin	Q9HD89	59.755	56.2	75	**0.56**
92	Annexin A2	P07355	36.573	25.5	23.1	**0.55**
93	Cell division control protein 42 homolog	P60953	28.922	56.9	57.7	**0.55**
94	Proteasome subunit alpha type-7	O14818	43.759	16.9	21	**0.54**
95	GTP-binding nuclear protein Ran	P62826	19.891	37	59.3	**0.54**
96	Aldehyde dehydrogenase	P30838	28.302	20.2	28.7	**0.54**
97	Kallikrein-1	P06870	10.689	32.7	20.8	**0.54**
98	Arylsulfatase A	P15289	11.608	37	37	**0.54**
99	Adenosylhomocysteinase	P23526	22.086	36.9	44.9	**0.54**
100	Plasminogen activator inhibitor 2	P05120	34.52	27.2	20.8	**0.53**
101	Translationally-controlled tumor protein	P13693	22.35	9	9	**0.53**
102	Complement factor H	P08603	596.33	21.1	20.8	**0.53**
103	Secreted Ly-6/uPAR-related protein 1	P55000	99.961	21	22.9	**0.53**
104	Prelamin-A/C;Lamin-A/C	P02545	36.431	19.8	14.3	**0.52**
105	Metalloproteinase inhibitor 1	Q5H9A7	11.771	32.8	42.2	**0.52**
106	Ig mu heavy chain disease protein	P04220	113.75	18.5	19.3	**0.52**
107	Vinculin	P18206	54.253	36.2	38.2	**0.51**
108	14-3-3 protein	P31946	76.631	18.3	27.3	**0.51**
109	Matrix metalloproteinase-9;67 kDa	P14780	32.922	28.3	31.1	**0.51**
110	Clusterin;Clusterin beta chain	P10909	77.969	74.3	78.4	**0.51**
111	Hemopexin	P02790	27.679	22.7	32.6	**0.51**
112	Ras-related protein Rap-1b	P61224	82.265	2.2	6.9	**0.51**
113	Granulins	P28799	65.059	10.2	5.2	**0.51**

Table 1e. Proteins with 2- 3-fold increased expression in the BMS sample relatively to healthy

no'	*Protein ID*	*Accession no'*	*Mol. weight [kDa]*	*Sequence coverage Healthy [%]*	*Sequence coverage BMS [%]*	*BMS/Healthy intensity ratio*
1	Lactoylglutathione lyase	Q04760	20.777	8.7	22.3	**1.9848**
2	Prominin-1	O43490	97.201	3.9	3.9	**1.9854**
3	Fructose-bisphosphate aldolase	P09972	39.455	22.8	18.7	**1.9878**
4		K7ERG3	19.306	37.6	46.5	**1.9921**
5	Ubiquitin-like modifier	P22314	117.85	7	13	**1.9964**
6	Guanine nucleotide-binding protein	P62873	12.285	16.7	33.3	**1.9981**
7	Interleukin-36 alpha	Q9UHA7	17.684	7.6	16.5	**2.0008**
8	Myeloid cell nuclear differentiation antigen	P41218	45.836	3.4	11.5	**2.0023**
9	Azurocidin	P20160	26.885	12.4	12.4	**2.0119**
10	Myosin light polypeptide 6	F8W1R7	16.29	33.8	33.8	**2.0165**
11	Heat shock 70 kDa protein	P08107	70.051	54.9	59.1	**2.0196**
12	Ig heavy chain V-I region V35	P23083	13.009	19.7	10.3	**2.0212**
13	Small proline-rich protein 3	Q9UBC9	17.342	45.3	64.6	**2.0244**
14	Galectin-3	P17931	26.152	10.4	20.4	**2.0369**
15	Collagen alpha-1(VI) chain	P12109	108.53	2.8	4.7	**2.0452**
16	Protein AMBP	P02760	38.999	19.9	13.9	**2.0457**
17	Immunoglobulin J chain	P01591	18.098	66.7	78.6	**2.0485**
18	Ig kappa chain V-I region WEA	P01610	11.84	36.1	36.1	**2.0504**
19	Ubiquitin-60S	P62979	10.469	45.2	57	**2.0540**
20	Actin, cytoplasmic 1	P60709	41.736	81.1	78.4	**2.0592**
21	Ras-related protein Rab-1A	P62820	19.018	9.8	6.4	**2.0832**
22	Apolipoprotein D	P05090	21.275	9.5	15.3	**2.0871**
23	Histidine-rich glycoprotein	P04196	59.578	7.4	13.5	**2.0926**
24	Serum amyloid	P02743	25.387	16.6	12.1	**2.0975**
25	Cathepsin S	P25774	37.495	2.4	2.4	**2.0976**
26	Dipeptidyl peptidase 3	Q9NY33	82.588	6.8	8.5	**2.0979**
27	Calcineurin-like phosphoesterase	Q9BRF8	35.548	6.4	21.3	**2.0984**
28	Cadherin-1;E-Cad	P12830	90.941	5	10.6	**2.1236**
29	Beta-2-glycoprotein 1	P02749	38.298	19.7	31	**2.1465**
30	Beta-hexosaminidase	P06865	58.44	6.3	15.1	**2.1479**
31	Keratin, type II cytoskeletal 6B	P04259	60.066	40.4	41.5	**2.1496**
32	Transmembrane protease serine 11D	O60235	32.933	33.6	47.2	**2.1579**
33	Beta-2-microglobulin	P61769	13.714	26.1	72.3	**2.1655**
34	Rho GDP-dissociation inhibitor 2	P52566	22.988	60.2	77.1	**2.1665**
35	Leukotriene A-4 hydrolase	P09960	69.284	36.3	54	**2.1675**

36	Extracellular matrix protein 1	Q16610	60.673	12.4	20.9	**2.1743**
37	Malate dehydrogenase, cytoplasmic	P40925	36.426	32.9	44	**2.1765**
38	Lactoperoxidase	P22079	73.956	38.7	62.6	**2.1785**
39	SH3 domain-binding glutamic acid-rich-like protein	O75368	12.774	26.3	48.2	**2.1799**
40	RNA binding motif protein, X-linked-like-1	P38159	8.6098	23.1	39.7	**2.1883**
41	Haptoglobin	P00738	45.205	22.4	51.5	**2.1949**
42	Ig kappa chain V-III region VG	P04433	12.575	32.2	58.3	**2.2047**
43	Perilipin-3	O60664	47.074	6.5	9.2	**2.2154**
44	BPI fold-containing family B member 2	Q8N4F0	49.172	26.9	34.3	**2.2177**
45	Hemoglobin subunit beta	P68871	15.998	89.1	79.6	**2.2194**
46	Elafin	P19957	12.269	18.8	28.2	**2.2262**
47	Melanotransferrin	P08582	80.214	7	9.6	**2.2271**
48	Polymeric immunoglobulin receptor;Secretory component	P01833	83.283	63.5	61	**2.2431**
49	Annexin A3;Annexin	P12429	36.375	20.4	32.2	**2.2529**
50	Lysozyme C	P61626	16.537	61.5	57.4	**2.2558**
51	Monocyte differentiation antigen CD14	P08571	40.076	32.8	35.5	**2.2789**
52	Trefoil factor 3	Q07654	8.641	31.2	45	**2.2925**
53	Phosphoglycerate kinase 1	P00558	44.614	63.8	79.4	**2.3140**
54	F-actin-capping protein subunit beta	P47756	31.35	13.4	36.1	**2.3213**
55	Alpha-2-macroglobulin-	A8K2U0	161.1	30.7	43.1	**2.3395**
56	Antileukoproteinase	P03973	14.326	17.4	17.4	**2.3431**
57	Cystatin-S	P01036	16.214	80.1	77.3	**2.3488**
58	Alpha-amylase 1	P04745	57.767	68.3	72.8	**2.3568**
59	Peroxiredoxin-4	Q13162	30.54	15.5	38	**2.3622**
60	Glutathione S-transferase omega-1	P78417	27.566	22.8	46.1	**2.3722**
61	Complement C4-A	P0C0L4	192.78	3.2	7.6	**2.4014**
62	Inter-alpha-trypsin inhibitor heavy chain H2	P19823	105.21	12.1	15.8	**2.4289**
63	Apolipoprotein A-IV	P06727	45.398	3	8.1	**2.4335**
64	Ig kappa chain C region	P01834	11.609	100	97.2	**2.4359**
65	Ras-related protein Rab-11A	P62491	17.621	27.7	45.2	**2.4466**
66	Ig alpha-2 chain C region	P01877	36.526	66.8	74.7	**2.4716**
67	Mesothelin	Q13421	37.561	7.2	19.5	**2.4965**
68	Ig kappa chain V-I region Ka	P01603	11.285	11.1	11.1	**2.5035**
69	Prolactin-inducible protein	P12273	16.572	70.5	77.4	**2.5045**
70	Lysosomal alpha-mannosidase	O00754	113.74	1.6	2.9	**2.5158**
71	Protein S100-A4	P26447	11.728	42.6	49.5	**2.5358**
72	Proteasome subunit alpha type-2	P25787	25.898	15.4	21.4	**2.5490**
73	Proteasome subunit beta type-1	P20618	26.489	14.5	30.7	**2.5546**
74	Ig kappa chain V-IV region	P01625	12.64	36.8	36.8	**2.5583**
75	Peptidyl-glycine alpha-amidating	P19021	108.33	6.3	8.1	**2.5690**
76	Neutral alpha-glucosidase AB	Q14697	106.87	6.5	8.9	**2.5785**
77	Putative elongation factor	Q5VTE0	50.184	4.5	8.4	**2.5799**

78	Serum albumin	P02768	69.366	75.9	82.1	**2.5870**
79	Carbonic anhydrase 6	P23280	35.366	49	66.6	**2.5990**
80	Purine nucleoside phosphorylase	P00491	32.118	26.6	45.7	**2.6050**
81	Liver carboxylesterase 1	P23141	62.52	13.2	21.3	**2.6383**
82	Profilin-1	P07737	15.054	70.7	97.1	**2.6441**
83	14-3-3 protein epsilon	P62258	29.174	32.5	42	**2.6505**
84	Inter-alpha-trypsin inhibitor	P19827	101.39	5.7	11.7	**2.6518**
85	Endoplasmic reticulum resident protein 29	P30040	28.993	8.4	8.4	**2.6589**
86	Cathepsin G	P08311	28.837	18.8	35.3	**2.6612**
87	Alpha-2-HS-glycoprotein	P02765	39.324	18.8	26.2	**2.6747**
88	Kininogen-1	P01042	71.957	13.2	25.9	**2.6960**
89	Mucin-1	P15941	28.297	4.2	8.5	**2.7026**
90	Enolase-phosphatase E1	Q9UHY7	19.187	8.1	12.7	**2.7210**
91	Superoxide dismutase	P04179	19.73	31.2	26.7	**2.7284**
92	Zymogen granule protein 16	Q96DA0	22.739	16.8	31.2	**2.7341**
93	Keratin, type I cytoskeletal 14	P02533	51.561	43.6	45.3	**2.7954**
94	Alpha-amylase 2B	P19961	57.709	60.1	57.7	**2.8022**
95	Dermcidin	P81605	11.284	30	36.4	**2.8144**
96	Ig lambda chain V-III region LOI	P80748	11.935	23.4	37.8	**2.8232**
97	Calmodulin-like protein 5	Q9NZT1	15.892	58.9	78.1	**2.8692**
98	Alpha-actinin-4	O43707	104.85	23.7	59.8	**2.9038**
99	Galectin-3-binding protein	Q08380	65.33	34.2	40.7	**2.9400**
100	Ig mu chain C region	P01871	49.306	46.9	54.4	**2.9731**
101	Actin-related protein 2/3 complex	P59998	19.667	6	12.5	**2.9834**

Table 1f. 158 Proteins with more than 3-fold increased expression in the BMS sample relatively to Healthy group

no'	*Protein ID*	*Accession no'*	*Mol. weight [kDa]*	*Sequence coverage Healthy [%]*	*Sequence coverage BMS [%]*	*BMS/Healthy intensity ratio*
1	Protein S100-A9	P06702	13.242	69.3	86	**2.9956**
2	Protein CutA	O60888	19.116	17.9	33	**3.0322**
3	Complement factor B	P00751	140.94	17	20.5	**3.0626**
4	Ig heavy chain V-I region HG3	P01743	12.946	18.8	22.2	**3.1005**
5	Complement C1s	P09871	76.684	1.2	2.8	**3.1190**
6	6-phosphogluconate dehydrogenase	P52209	51.872	22.1	47	**3.1379**
7	Protein S100-A8	P05109	10.834	60.2	60.2	**3.1493**
8	Arginase-1	P05089	34.735	12.4	45.7	**3.1498**
9	L-lactate dehydrogenase A chain	P00338	36.688	42.8	54.2	**3.1577**
10	Annexin A1	P04083	38.714	35.8	51.7	**3.1684**
11	Periplakin	O60437	131.37	0.9	2.6	**3.1736**
12	Echinoderm microtubule-associated	O95834	70.678	2.9	5.4	**3.1885**
13	Vitronectin	P04004	54.305	13.8	17.8	**3.2126**
14	Alpha-actinin-1	P12814	103.06	27.8	48.1	**3.2286**
15	Actin, cytoplasmic 2	P63261	41.792	81.1	78.4	**3.2609**
16	Actin-related protein 2/3 complex	O15143	40.949	2.7	6.7	**3.2847**
17	Plastin-2	P13796	70.288	34.1	51	**3.3064**
18	Apolipoprotein B-100	P04114	515.6	0.8	1.3	**3.3086**
19	Cystatin-A	P01040	11.006	27.6	76.5	**3.3197**
20	BPI fold-containing family B member 1	Q8TDL5	52.441	8.7	14.9	**3.3391**
21	Ig lambda chain V-II region NIG-84	P04209	11.581	8	8	**3.3403**
22	Filamin-A	P21333	280.74	3	10.7	**3.3685**
23	Ig gamma-3 chain C region	P01860	41.287	23.6	41.4	**3.3687**
24	Adenylosuccinate synthetase isozyme 2	P30520	50.097	3.1	5.9	**3.3833**
25	Cytochrome c	P99999	11.333	22.8	34.7	**3.4251**
26	Leukocyte elastase inhibitor	P30740	42.741	36.7	53	**3.4475**
27	N-acetylglucosamine-6-sulfatase	P15586	56.374	2.4	2.8	**3.5038**
28	Myosin-9	P35579	226.53	2.1	5.5	**3.5336**
29	Ly6/PLAUR domain-containing protein 3	O95274	35.97	27.7	30.6	**3.5661**
30	Serpin B13	Q9UIV8	44.276	22.5	56.3	**3.5977**
31	Afamin	P43652	69.068	19.5	37.6	**3.6116**
32	UPF0762 protein C6orf58	Q6P5S2	37.926	50	53.9	**3.6417**
33	Inter-alpha-trypsin inhibitor heavy chain	Q14624	103.36	11	21.8	**3.6431**
34	Fibrinogen beta chain	P02675	55.928	6.9	11.4	**3.6461**
35	WAP four-disulfide core domain protein	Q14508	12.993	45.2	45.2	**3.6821**
36	Adenylyl cyclase-associated protein 1	Q01518	51.901	26.1	59.4	**3.6935**
37	Ig kappa chain V-II region RPMI 6410	P06310	14.707	18	37.6	**3.6976**
38	Angiotensinogen;Angiotensin-1	P01019	53.154	10.7	21.2	**3.7031**
39	Ig gamma-2 chain C region	P01859	35.9	25.2	32.5	**3.7035**

40	IgGFc-binding protein	Q9Y6R7	572.01	7.8	16.7	**3.7100**
41	Apolipoprotein M	O95445	21.253	4.8	8.5	**3.7971**
42	Complement C3	P01024	187.15	10.7	31.2	**3.8092**
43	Ig kappa chain V-I region Ni	P01613	11.745	30.4	30.4	**3.8165**
44	Ceruloplasmin	P00450	122.2	7.5	20.4	**3.8264**
45	Acyl-CoA-binding protein	P07108	10.044	59.8	72.4	**3.8353**
46	Lipocalin-1	P31025	19.25	73.9	68.2	**3.8362**
47	Transcobalamin-1	P20061	48.206	34.4	32.1	**3.8380**
48	Ig lambda-2 chain C regions	P0CG05	11.293	96.2	96.2	**3.8397**
49	Keratin, type I cytoskeletal 16	P08779	51.267	47.6	36.6	**3.8433**
50	Ras GTPase-activating-like protein IQGAP1	P46940	189.25	3.2	2.7	**3.8859**
51	Peptidyl-prolyl cis-trans isomerase B	P23284	23.742	11.6	29.6	**3.9194**
52	Elongation factor 2	P13639	95.337	1.6	8.7	**3.9614**
53	Acylamino-acid-releasing enzyme	P13798	81.224	1.6	4.8	**3.9643**
54	Fascin	Q16658	52.262	2.1	2.1	**3.9851**
55	Transforming protein RhoA	P61586	21.768	14	19.2	**4.0074**
56	Calpain-1 catalytic subunit	P07384	81.889	4.8	7.4	**4.0133**
57	Cystatin-B	P04080	11.139	82.7	91.8	**4.0191**
58	Ig kappa chain V-I region EU	P01598	11.788	32.4	32.4	**4.0438**
59	Maltase-glucoamylase,	O43451	209.85	1.5	4.3	**4.0527**
60	Ig heavy chain V-II region	P04438	16.323	4.8	10.9	**4.0597**
61	Glutathione peroxidase 3	P22352	25.57	11.1	16.4	**4.0603**
62	Protein S100-A12;Calcitermin	P80511	10.575	41.3	48.9	**4.0874**
63	Proactivator polypeptide	P07602	58.112	13.7	28.8	**4.1827**
64	Protein S100-P	P25815	10.4	34.7	35.8	**4.1980**
65	Glycogen phosphorylase, liver form;Phosphorylase	P06737	97.147	5.3	12.2	**4.2028**
66	Hexokinase-3	P52790	99.024	1.3	4	**4.2048**
67	Folate receptor gamma	P41439	27.638	15.6	35.4	**4.2070**
68	Heterogeneous nuclear ribonucleoproteins A2/B1	P22626	37.429	5.7	9.6	**4.3215**
69	Myeloblastin	P24158	27.807	20.3	23.4	**4.3608**
70	Thyroxine-binding globulin	P05543	46.324	8.9	17.3	**4.3843**
71	Alpha-2-macroglobulin	P01023	163.29	17.2	39.1	**4.4315**
72	Glucose-6-phosphate isomerase	P06744	63.146	27.6	51.4	**4.4482**
73	Ezrin	P15311	69.412	13.8	40.8	**4.4521**
74	Serpin B3	P29508	44.564	45.6	70.5	**4.4717**
75	Nicotinate phosphoribosyltransferase	Q6XQN6	57.578	2.6	5	**4.5008**
76	Antithrombin-III	P01008	52.602	17.7	27.4	**4.5416**
77	Histone H1.4	P10412	21.865	9.1	9.6	**4.5948**
78	Hypoxanthine-guanine phosphoribosyltransferase	P00492	24.579	9.6	12.8	**4.6019**
79	Glycogenin-1	P46976	21.51	7.3	15.5	**4.6377**
80	Fibrinogen alpha chain	P02671	94.972	4.6	9.2	**4.6665**
81	Moesin	P26038	67.819	16.5	50.1	**4.6891**
82	Integrin beta;Integrin beta-2	P05107	78.345	2.1	3.4	**4.7118**
83	UTP--glucose-1-phosphate uridylyltransferase	Q16851	56.94	1.8	1.8	**4.7475**
84	Protein S100-A7	P31151	11.471	38.6	71.3	**4.7544**

85	Ubiquitin-conjugating enzyme E2 N	P61088	17.138	7.2	18.4	**4.7692**
86	Ig gamma-1 chain C region	P01857	36.105	34.8	55.8	**4.7887**
87	Ras-related protein Rab-7a	P51149	23.489	4.8	30.9	**4.7921**
88	Serpin B5	P36952	42.1	5.6	16	**4.7963**
89	Hepatoma-derived growth factor	P51858	26.788	5.8	12.9	**4.8847**
90	Actin-related protein 2/3 complex	O15144	34.333	6.3	36	**4.9000**
91	Rho GDP-dissociation inhibitor 1	P52565	23.207	34.8	55.4	**5.0771**
92		P13646	49.586	43	40.8	**5.1168**
93	Aspartate aminotransferase	P00505	47.517	8.1	8.8	**5.1523**
94	Serotransferrin	P02787	77.063	34.4	60.7	**5.2887**
95	Keratin, type II cytoskeletal 1	P04264	66.038	33.4	56.7	**5.3278**
96	NAD(P)H-hydrate epimerase	Q8NCW5	31.674	3.1	7.3	**5.3524**
97	Keratin, type I cytoskeletal 17	Q04695	48.105	26.9	33.6	**5.4253**
98	Keratin, type II cytoskeletal 2 oral	Q01546	65.84	15	16.8	**5.4278**
99	Alpha-1-acid glycoprotein 2	P19652	23.602	7.5	11.9	**5.5272**
100	Aldo-keto reductase	O60218	36.019	18	29.4	**5.6093**
101	Neutrophil collagenase	P22894	53.411	16.5	30.4	**5.6783**
102	Serpin B10	P48595	45.402	7.3	33.2	**5.8505**
103	Keratin, type II cytoskeletal 6C	P48668	60.024	45.6	41.5	**5.9981**
104	Apolipoprotein A-II	P02652	11.175	10	22	**6.0239**
105	Attractin	O75882	158.54	1.6	3.6	**6.0290**
106	Ig heavy chain V-III region VH26	P01764	12.582	18.8	44.4	**6.0832**
107	Puromycin-sensitive aminopeptidase	P55786	102.99	5.1	19.6	**6.1107**
108	Serpin B8;Serpin B9	P50452	42.766	5.9	5.9	**6.1789**
109	Cystatin-D	P28325	16.08	71.8	71.8	**6.2248**
110	Thymosin beta-4	P62328	5.0526	86.4	86.4	**6.2757**
111	Calreticulin	P27797	48.141	7.9	12	**6.3207**
112	Ig lambda chain V-I region WAH	P04208	11.725	11.9	27.5	**6.4303**
113	Glucose-6-phosphate 1-dehydrogenase	P11413	59.256	15.5	37.7	**6.4559**
114	Calmodulin-like protein 3	P27482	16.891	4.7	34.2	**6.4689**
115	Transforming growth factor	Q15582	39.793	4.9	4.9	**6.5314**
116	Vasodilator-stimulated phosphoprotein	P50552	39.829	3.7	9.5	**6.5946**
117	Cornifin-A	P35321	9.8774	61.8	61.8	**6.6586**
118	Epididymal secretory protein E1	P61916	16.23	33.8	44.6	**6.9151**
119	Translin-associated protein X	Q99598	21.427	10.8	22.6	**6.9256**
120	Keratin, type I cytoskeletal 9	P35527	62.064	16.2	57	**6.9957**
121	Phosphoglucomutase-2	Q96G03	68.283	2.9	14.2	**7.0844**
122	Keratin, type I cytoskeletal 10	P13645	58.826	27.6	60.6	**7.3539**
123	Neutrophil gelatinase-associated lipocalin	P80188	22.588	38.4	75.3	**7.3723**
124	Sulfhydryl oxidase 1	O00391	82.577	2	7.5	**7.3849**
125	Phosphoglucomutase-1	P36871	61.448	6	18.3	**7.3943**
126	Keratin, type II cytoskeletal 4	P19013	57.285	38.4	37.1	**7.5871**
127	Tyrosine-protein phosphatase	P78324	54.966	3.2	5.8	**7.7211**
128	Talin-1	Q9Y490	258.08	0.5	3.6	**7.9036**
129	Chitinase-3-like protein 1	P36222	42.625	6.3	17	**8.3726**
130	Endoplasmic reticulum resident protein	Q9BS26	46.971	4.4	10.1	**8.5032**
131	Non-secretory ribonuclease	P10153	18.354	10.6	23	**8.6851**

132	Serine protease inhibitor Kazal-type 7	P58062	6.5625	15.3	76.3	**8.8977**
133	Proteasome subunit beta type-3	P49720	16.161	8.3	24.1	**9.2131**
134	Costars family protein ABRACL	Q9P1F3	9.0564	19.8	46.9	**9.4789**
135	Keratin, type II cytoskeletal 5	P13647	62.378	28.3	44.2	**9.6575**
136	Carcinoembryonic antigen-related cell	P31997	38.153	9.7	23.8	**9.8336**
137	Ig gamma-4 chain C region	P01861	35.94	21.1	36.4	**10.3679**
138	SPARC-like protein 1	Q14515	61.759	10.8	21.5	**10.3681**
139	Nucleobindin-2;Nesfatin-1	P80303	50.195	19	40.2	**10.4582**
140	Tubulin alpha-1B chain	P68363	50.151	7.1	5.3	**10.8572**
141	Protein S100-A11	P31949	11.74	41	86.7	**11.2862**
142		Q5VU59	27.174	49.6	56	**11.6189**
143	Keratin, type II cytoskeletal 2 epidermal	P35908	65.432	23.5	54	**12.3088**
144	Kallikrein-13	Q9UKR3	30.57	5.8	31.4	**12.9681**
145	Alpha-1-antichymotrypsin	P01011	47.65	2.4	10.6	**13.3975**
146	Phospholipid transfer protein	P55058	54.739	2.2	5.3	**14.4994**
147	Beta-glucuronidase	P08236	58.345	1.6	9.7	**16.4347**
148	Immunoglobulin lambda-like polypeptide	P0CG04	23.063	51.4	51.4	**16.6113**
149	Protein disulfide-isomerase A6	Q15084	47.837	2.1	14.6	**17.0145**
150	Alpha-1-acid glycoprotein 1	P02763	23.511	9	11.9	**17.2960**
151	Proteasome activator complex subunit 2	Q9UL46	26.011	7.9	17.1	**17.9050**
152	Lymphocyte antigen 6D	Q14210	13.286	10.9	18.8	**19.9674**
153		Q5RHS7	10.97	17.9	41.1	**22.9774**
154	Heme-binding protein 2	Q9Y5Z4	22.875	4.9	41.5	**23.7018**
155	Keratin, type II cuticular Hb4	Q9NSB2	64.842	6.3	8.3	**25.7195**
156	Protein S100-A6	P06703	9.681	9.4	62.4	**65.4174**
157	Fumarylacetoacetase	P16930	46.374	3.8	22.9	**88.8516**
158	Dipeptidyl peptidase 4	P27487	88.278	1	8.6	**129.8391**

Table 1g. Proteins identified only in BMS

no'	*Protein ID*	*Accession no'*	*Mol. weight [kDa]*	*Sequence coverage Healthy [%]*	*Sequence coverage BMS [%]*	*BMS/ Healthy intensity ratio*
1	L-selectin	P14151	42.187	0	2.4	**4900**
2	Haloacid dehalogenase-like hydrolase	Q9H0R4	22.49	0	6.4	**9468**
3	Ig delta chain C region	P01880	42.253	0	2.1	**11191**
4	Annexin	P08133	75.276	0	1.5	**11488**
5	Heat shock protein 90-beta	P08238	83.263	1.7	3.5	**14400**
6	Phosphoacetylglucosamine mutase	O95394	59.851	0	1.5	**15522**
7	Histone H2A type 1-C	Q93077	14.105	5.4	13.8	**27880**
8	Prostaglandin reductase 1	Q14914	13.444	0	9.3	**31183**
9	T-complex protein 1 subunit beta	P78371	44.812	0	2.6	**31921**
10	Dihydrolipoyl dehydrogenase	P09622	43.587	0	2.7	**34708**
11	CD5 antigen-like	O43866	38.087	0	4.6	**34857**
12	Serine/threonine-protein phosphatase	P62140	37.186	0	3.4	**35094**
13	Protein-arginine deiminase type-4	Q9UM07	74.078	0	2.7	**35268**
14	Grancalcin	P28676	16.712	0	8.2	**35594**
15	Ferritin light chain	P02792	20.019	0	9.1	**35932**
16	Hepatocyte growth factor	Q04756	70.681	0	2.1	**36297**
17	Bridging integrator 2	Q9UBW5	59.186	0	1.9	**36614**
18	Filamin-B	O75369	256.28	0	0.5	**37789**
19	Translin	Q15631	25.572	0	5.8	**44693**
20	Insulin-like growth factor-binding protein	P35858	66.034	0	2.3	**46647**
21	Haptoglobin-related protein	P00739	39.029	4.9	17.2	**54138**
22	Complement factor H-related protein 2	P36980	30.65	0	3.7	**57667**
23	Integrin alpha-M	P11215	127.18	0	1	**63242**
24	Plasminogen	P00747	90.568	0	1.2	**74309**
25	Glia maturation factor gamma	O60234	16.801	0	11.3	**74356**
26	Heparin cofactor 2	P05546	57.07	0	3	**76225**
27		D6RF35	53.02	58.8	64.7	**76984**
28	Elongation factor 1-gamma	P26641	50.118	0	4.6	**77305**
29	DNA damage-binding protein 1	Q16531	126.97	0	1.1	**78905**
30	Protein XRP2	O75695	39.641	0	2.3	**82118**
31	Malic enzyme	P48163	55.165	0	3.2	**84360**
32	Inter-alpha-trypsin inhibitor heavy chain	Q06033	99.848	0	2.5	**87499**
33	Xaa-Pro dipeptidase	P12955	54.548	0	2	**90249**
34	Apolipoprotein E	P02649	36.154	0	6.3	**90607**
35	Complement component C6	P13671	104.79	0	1.2	**93437**
36		F5GWP8	66.35	15.6	22.5	**93451**

37	Tubulin beta-4B chain	P68371	49.83	0	4.3	**94868**
38	4-trimethylaminobutyraldehyde dehydrogenase	P49189	53.801	0	5.5	**96844**
39	Protein FAM49B;Protein FAM49A	Q9NUQ9	20.193	0	7.3	**97539**
40	Plasma kallikrein	P03952	71.369	0	1.6	**103420**
41	Oligoribonuclease, mitochondrial	Q9Y3B8	13.472	0	14.4	**106300**
42	Ribonuclease inhibitor	P13489	49.973	0	2.2	**110160**
43	Protein canopy homolog 2	Q9Y2B0	19.052	0	9.5	**110670**
44	14-3-3 protein eta	Q04917	28.218	5.7	14.2	**113490**
45	cAMP-dependent protein kinase type I	P10644	33.606	0	4	**120620**
46	Keratin, type II cytoskeletal 78	Q8N1N4	56.865	3.8	8.8	**120700**
47	1,4-alpha-glucan-branching enzyme	Q04446	80.473	0	4.4	**125440**
48	Rab GDP dissociation inhibitor alpha	P31150	50.582	4.9	15.9	**126590**
49	Coronin-1C	Q9ULV4	53.248	0	2.5	**129260**
50	Glyoxylate reductase	Q9UBQ7	35.668	0	8.8	**130710**
51	Proteasome subunit beta type-8	P28062	30.354	0	4	**131980**
52	CD177 antigen	Q8N6Q3	46.363	0	2.5	**133790**
53	Ubiquitin carboxyl-terminal hydrolase	P54578	51.086	0	4.7	**145160**
54	Aminopeptidase N	P15144	109.54	0	2.1	**147820**
55	Ig heavy chain V-II region WAH	P01824	14.117	0	6.2	**149370**
56	Actin-related protein 2	P61160	44.76	0	5.6	**151130**
57	Complement component C9	P02748	63.173	0	5.5	**164960**
58	Plasma protease C1 inhibitor	P05155	49.757	0	5.6	**168400**
59	Glucosamine-6-phosphate isomerase 1	P46926	30.9	0	18.2	**171770**
60	Apolipoprotein C-I	P02654	8.647	0	26	**171780**
61	Complement C5	P01031	188.3	0	1.7	**175430**
62	N-acetyl-D-glucosamine kinase	Q9UJ70	37.375	0	4.9	**175760**
63	Brain acid soluble protein 1	P80723	22.693	0	22.9	**180630**
64	Junction plakoglobin	P14923	81.744	0	5.9	**191430**
65	Heterogeneous nuclear ribonucleoprotein	Q14103	29.724	0	6.5	**197760**
66	Farnesyl pyrophosphate synthase	P14324	48.275	4.3	4.3	**211570**
67	Mitochondrial peptide methionine sulfoxide reductase	Q9UJ68	26.132	0	12.8	**237270**
68	DNA-(apurinic or apyrimidinic site) lyase	P27695	35.554	0	8.2	**256100**
69	Dipeptidyl peptidase 2	Q9UHL4	54.341	0	5.3	**265840**
70	Proteasome subunit beta type-6	P28072	25.357	0	9.2	**266950**
71	Isocitrate dehydrogenase [NADP]	O75874	46.659	0	13.5	**270120**
72	Proteasome subunit alpha type	P25789	23.27	0	12.9	**273790**
73	Keratin, type I cytoskeletal 19	P08727	44.105	13	20.2	**285560**
74	Calpain small subunit 1	P04632	28.315	0	7.1	**291930**
75	Bactericidal permeability-increasing protein	P17213	53.899	0	10.3	**351610**
76	Lysosome-associated membrane glycoprotein 1	P11279	38.985	0	8.8	**352300**
77	Proteasome subunit beta type-4	P28070	29.204	0	14	**372860**

78	Small ubiquitin-related modifier 2	P61956	10.871	0	12.6	**398790**
79	Neutrophil cytosol factor 1	P14598	44.651	0	8.2	**418620**
80	Corticosteroid-binding globulin	P08185	45.14	0	12.8	**438410**
81	Carbonic anhydrase 2	P00918	29.246	0	6.2	**444970**
82	Actin-related protein 2/3 complex	O15145	20.546	0	15.2	**448670**
83	Acid ceramidase	Q13510	34.604	0	11.5	**448890**
84	Proteasome activator complex subunit 1	Q06323	28.723	0	7.6	**450080**
85	Lysosome-associated membrane glycoprotein 2	P13473	32.979	0	2.7	**507780**
86	UV excision repair protein	P54727	15.213	0	12.3	**548090**
87	Nicotinamide phosphoribosyltransferase	P43490	53.392	0	11.4	**681530**
88	Beta-mannosidase	O00462	94.411	0	7.7	**691900**
89	Hornerin	Q86YZ3	282.39	0	8	**696310**
90	Lysosomal Pro-X carboxypeptidase	P42785	55.799	0	11.9	**767070**
91	Suprabasin	Q6UWP8	60.54	9.2	15.3	**881340**
92	Heat shock 70 kDa protein 4	P34932	94.33	0	14.6	**949480**
93	Carbonic anhydrase 1	P00915	28.87	0	27.6	**1033900**
94	CD9 antigen	P21926	17.764	0	28.9	**1227000**
95	Fibrinogen gamma chain	P02679	50.322	0	13.9	**1256600**
96	Beta-hexosaminidase subunit beta	P07686	38.258	0	18.1	**1289300**
97	Annexin A5;Annexin	P08758	35.936	0	3.4	**1320700**
98	Protein S100-A2	P29034	11.117	0	39.8	**1330900**
99	Olfactomedin-4	Q6UX06	57.279	0	20.6	**1648400**
100	Calmodulin	P62158	16.837	4.7	34.9	**18485000**

Table S2: functional clusters division, presented in ratio groups

Ratio group d	*Sum*	*Ratio group e*	*Sum*	*Ratio group f*	*Sum*	*Ratio group g*	*Sum*
disulfide bond	83	signal	53	signal	60	signal	31
hydrolase	52	proteolysis	16	cytoskeleton	37	proteolysis	24
cytoskeleton	34	immune response	15	enzyme inhibitor activity	22	hydrolase	20
cytoplasmic membrane-bounded vesicle	33	response to wounding	12	calcium binding	20	plasma	11
regulation of apoptosis	30	membrane-bounded vesicle	12	cell motion	18	membranal proteins	11
homeostatic process	30	enzyme inhibitor activity	11	proteolysis	18	calcium binding	11
defense response	25	plasma	10	plasma	17	response to wounding	10
macromolecular complex assembly	25	negative regulation of programmed cell death	10	actin binding	14	transition metal ion binding	10
positive regulation of molecular function	21	carbohydrate binding	9	immune response	14	immune response	10
actin binding	19	cell fraction	9	homeostatic process	14	enzyme inhibitor activity	9
isopeptide bond	17	extracellular matrix	7	cell adhesion	13	cytoskeleton	9
lysosome	16	cellular homeostasis	7	keratin	12	complement pathway	8
carbohydrate catabolic process	16	response to bacterium	6	carbohydrate catabolic process	11	lipid binding	8
peptidase inhibitor activity	16	glycosidase	5	epithelial cell differentiation	10	oxidation reduction	8
Redox-active center	13	antioxidant activity	5	pyroglutamic acid	8	lysosome	7
response to temperature stimulus	13	vascular process in circulatory system	4	basolateral plasma membrane	8	sexual reproduction	6
proteolysis	11	lipoprotein particle	3	Pyrrolidone carboxylic acid	7	external side of plasma membrane	5
ectoderm development	11	positive regulation of endocytosis	3	Leukocyte migration	7	steroid metabolic process	5
endoplasmic reticulum part	10			protein processing	7	CCP	4
secretory granule	10			maintenance of location	6	negative regulation of coagulation	4
cytokine activity	9			polysaccharide catabolic process	5	regulation of proteolysis	4
myofibril	8					lipoprotein particle	3
membrane protein	6					Ubiquitin	3
14_3_3	5						
protease inhibitor	4						

Table S3: Sampling data of healthy and BMS groups

	Sample No.	*Gender*	*Age*	*Sialomerty (5 min)*	*Place of burning*
BMS pooling samples	1	F	50	3.3	all intraoral mucosa
	2	F	55	2.4	tongue
	3	F	83	2.1	tongue
	4	F	56	2.9	all intraoral mucosa
	5	F	61	2.6	tounge/lips
	6	F	60	3.1	tongue
	7	F	66	2.7	tongue
	8	F	61	2.9	tongue
	9	F	77	3.8	tongue
	10	F	64	3	tongue
	11	F	58	2.7	tongue
	12	F	81	2.5	tongue
	13	F	79	3	buccal, labial mucosa, tounge
	14	F	76	3.1	tongue
	15	F	68	2.8	tongue
	16	F	63	3.6	tongue
	17	F	70	4	tongue
	18	F	66	2.8	tongue
	19	F	68	3.1	tongue
	20	F	70	3.2	tongue
	N=20	***Average***	***66.6***	***3.0***	
		SD	***8.9***	***0.4***	
BMS individual validation samples	1	F	68	2.9	tongue
	2	F	73	3.3	tongue
	3	F	64	2.4	tongue
	4	F	72	3	tongue
	5	F	71	3	tongue
	N=5	***Average***	***69.6***	***2.9***	
		SD	***3.3***	***0.3***	
healthy pooling samples	1	F	69	3.2	
	2	F	84	3.6	
	3	F	58	2.4	
	4	F	66	2.7	
	5	F	59	2.4	
	6	F	71	4.3	
	7	F	77	4.5	
	8	F	75	3.1	
	9	F	65	3.5	
	10	F	67	3.5	
	11	F	72	2.7	
	12	F	56	3.9	
	13	F	80	3	
	14	F	55	2.8	
	15	F	60	3.3	
	16	F	92	3.9	
	17	F	78	1.7	
	18	F	68	2.2	
	19	F	74	2.6	

	20	F	76	4.3
	N=20	***Average***	***70.1***	***3.2***
		SD	***9.5***	***0.7***
healthy individual samples	1	F	66	2
	2	F	76	2.2
	3	F	71	3.6
	4	F	68	3
	5	F	69	3.1
	N=20	***Average***	***67.4***	***2.9***
		SD	***3.4***	***0.8***

Exclusion criteria

- *Pregnancy*
- *Folic acid, B12 deficiency*
- *Active infection*
- *Oral Lesions*
- *Diabetes*

Inclusion criteria:

- *Non-smoking female*
- *Healthy aged-matched control group*

Table S4: List of depleted HAP's

no'	*Protein*
1	Amylase
2	Albumin
3	α1-Acid Glycoprotein
4	IgG
5	Ceruloplasmin
6	IgA
7	Apolipoprotein A-I
8	IgM
9	Apolipoprotein A-II
10	IgD
11	Apolipoprotein B
12	Transferrin
13	Complement C1q
14	Fibrinogen
15	Complement C3
16	α2–Macroglobulin
17	Complement C4
18	α1-Antitrypsin
19	Plasminogen
20	Haptoglobin
21	Prealbumin
