# Supplementary material for: Proteomic profiling of whole-saliva reveals correlation between Burning Mouth Syndrome and the neurotrophin signaling pathway
Source: Sci Rep. 2019 Mar 18;9:4794. doi: 10.1038/s41598-019-41297-9 (PMC6423135; doi:10.1038/s41598-019-41297-9)

Proteomic profiling of whole-saliva reveals correlation between Burning Mouth Syndrome and the neurotrophin signaling pathway

Guy Krief[1,3], Yaron Haviv[2*], Omer Deutsch[1,3], Naama Keshet[2], Galit Almoznino[2]

Batia Zacks[2], Aaron Palmon[2], Doron J. Aframian[1]

Figure 5s. Protein association network

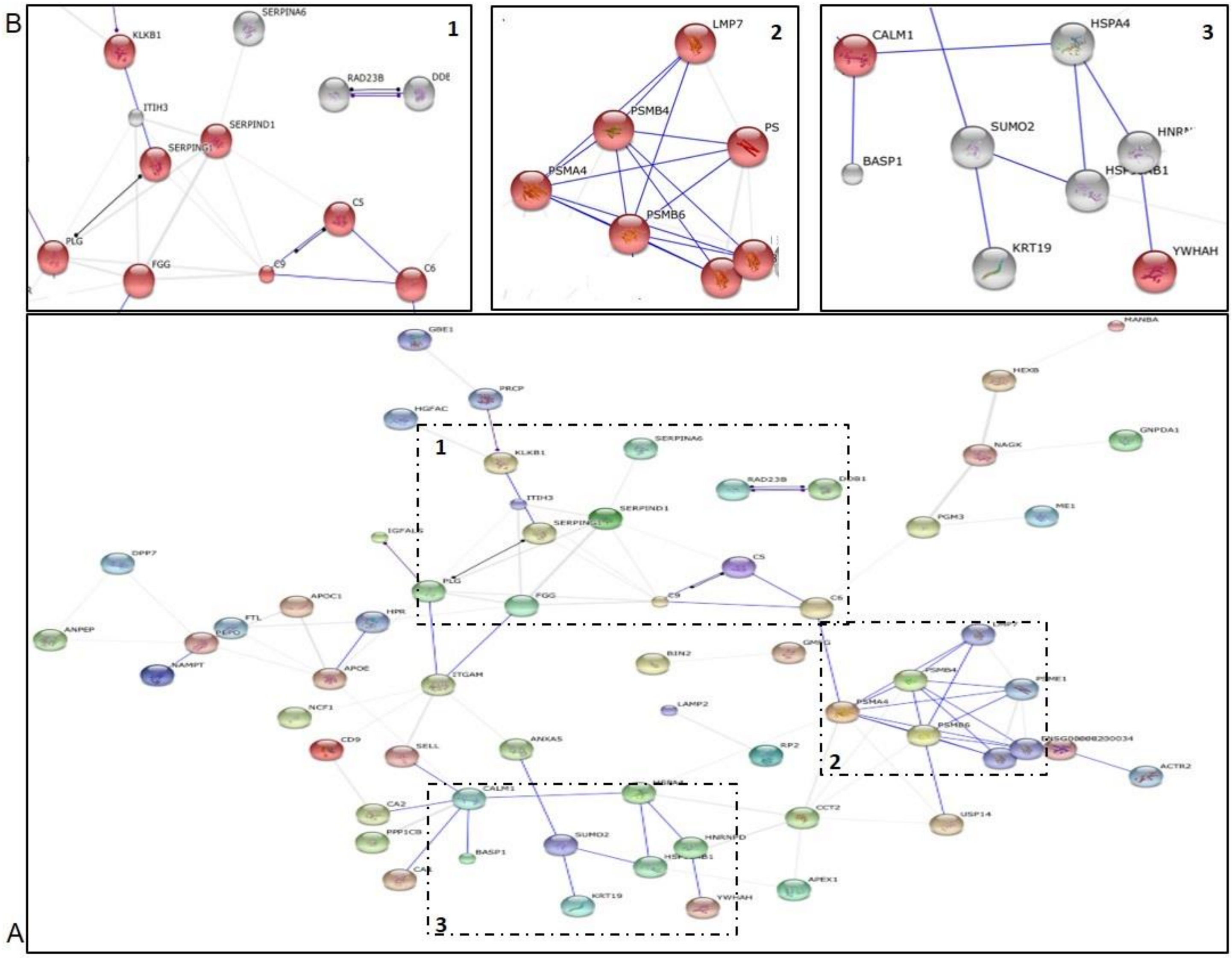

Supplement: Supplementary file 2 — Figure 5s. Protein association network [file 41598_2019_41297_MOESM2_ESM.pdf]
